# Effect of arginine supplementation during lactation on piglet survivability

**DOI:** 10.1093/tas/txag064

**Published:** 2026-05-19

**Authors:** Sydney E Craig, David A Clizer, Keith D Haydon, Laura L Greiner

**Affiliations:** Department of Animal Sciences, Iowa State University, Ames, IA, 50011, United States; CJ America—Bio, Fort Dodge, IA, 50501, United States; CJ America—Bio, Fort Dodge, IA, 50501, United States; Department of Animal Sciences, Iowa State University, Ames, IA, 50011, United States

**Keywords:** arginine, lactation, piglet mortality, sows

## Abstract

Two hundred and thirty-two PIC (PIC 1050, Genus, Hendersonville, TN) sows were used to investigate the effects of arginine supplementation on lactating sow performance and piglet mortality. Sows were allotted by randomized complete block design to one of two dietary treatments formulated to different standard ileal digestible (SID) arginine levels upon entering the farrowing. The control (CON) diet contained 0.95% SID arginine, while the experimental diet contained 1.12% SID arginine (ARG). Diets were formulated to meet or exceed all other NRC (2012) nutrient recommendations. Sow body weight (BW), backfat (BF), and body condition score (BCS) were recorded upon entering the farrowing room and upon weaning. Piglet litter weights were recorded after cross-fostering and at 20 days of age, to assess litter growth rate. Data were analyzed using generalized linear mixed models, with treatment as a fixed effect and group as a random effect. Post-foster litter size and litter weight were added as covariates. Models were fit using R (v4.4.1), and results were considered significant at *P* ≤ 0.05 and a tendency at 0.50 < *P* ≤ 0.10. Sow and litter were considered the experimental unit. Sow lactation length and BW at wean were not different between treatments (*P* = 0.76 and 0.63, respectively). There was no difference in ADFI with ARG consuming 7.28 ± 0.095 kg/d and the CON consuming 7.48 ± 0.095 kg/d (*P* = 0.12). The supplementation of Arg did not change BCS at weaning (2.72, 2.76 (scale of 1–5); *P* = 0.57). Upon entering the farrowing room, sow BF was 10.13 and 10.00 mm for the CON and Arg diets, respectively. The change in BF after weaning did not differ between dietary treatments (*P* = 0.53). The number of pigs weaned per sow was not different between sows in both treatments (12.1 ± 0.119 piglets, *P* = 0.79). Starting litter weight was 20.07 ± 0.357 kg for CON and 21.66 ± 0.357 kg for ARG. Litter wean weight did not differ between treatments (*P* = 0.69). Piglet average daily gain (ADG) differed significantly between treatments, with a mean of 0.25 ± 0.004 kg/d for CON and 0.24 ± 0.004 kg/d for ARG (*P* = 0.02). Post-foster mortality was not affected by Arg supplementation, with CON post-foster mortality at 6.37% and ARG at 5.96% (*P* = 0.43). This resulted in total livability of 93.3% (CON) and 94.0% (ARG), which did not differ (*P* = 0.43). In summary, Arg supplementation during lactation did not significantly influence sow performance or overall piglet growth and livability.

## Teaser

Improving piglet livability and sow performance remains a priority in modern swine production. Previous work has shown improvements in livability and wean weights when arginine is supplemented to sows during lactation. This study investigated the supplementation of arginine during lactation on piglet survivability. Results indicate that arginine supplementation does not impact piglet survivability.

## Introduction

Arginine (Arg) is a conditionally essential amino acid, defined as an amino acid that becomes essential when endogenous synthesis is insufficient to meet the animal’s requirements (Lewis and Southern 2001). Conditionality can occur during times of illness or stress, or inadequate dietary supply (Lewis and Southern 2001; NRC2012). Arginine plays a central role in multiple physiological processes, serving as a precursor for nitric oxide and polyamines, which are vital for vasodilation, immune function, and cell proliferation (Palmer et al. 1988; Flynn et al. 2002). Nitric oxide, in particular, is critical for vasodilation and increased blood flow, which can enhance milk production and mammary development, thereby improving nutrient availability (Trottier et al. 1997).

In addition to its role in vascular function, Arg contributes to immune function and tissue repair (Martí I Líndez and Reith 2021), further highlighting its importance during physiological stress or periods of rapid growth, such as farrowing and weaning. While endogenous synthesis is generally considered sufficient to meet the needs of the gestating sow, endogenous synthesis of young pigs is inadequate to support the growth in pre-wean pigs (Wu et al. 2004). Enterocyte Arg synthesis in 7-day old piglets is 75–88% lower than in newborn piglets (Wu 1997). Consequently, reduced intestinal Arg synthesis capacity diminishes nitric oxide synthesis (Wu and Meininger 2000). Arginine intake from sows’ milk does not meet the piglets’ needs for the pre-wean piglets’ rate of protein deposition, and it is estimated that sows’ milk provides less than 40% of the Arg requirement of a 7-day-old suckling pig (Wu et al. 2004). This is caused by arginase activity in the mammary gland, resulting in degradation of Arg and causing less output than uptake (Wu et al. 2007). Supplementing Arg above the National Research Council: Nutrient Requirements of Swine (NRC2012) recommendation may help the sow meet her needs and provide Arg for incorporation into milk protein for piglets.

Previous work supplemented Arg during lactation with total Arg 1.24% and found that piglets from supplemented sows had a higher bodyweight at wean, and maintained a higher bodyweight post-wean (Wessels et al. 2023). Luise et al. (2023) supplemented sows with 22.5 g Arg per day (SID Arg estimated 1.19%) and had lower mortality in piglets from supplemented sows, but no effects on sow bodyweight or litter weight. Zhu et al. (2017) supplemented sows with total Arg at 1.64% (SID Arg estimated at 1.53%) and observed improved litter weight gain. However, work by Holanda et al. (2019) did not see improved weights or mortality in pigs from sows supplemented with SID Arg 2.15%, this supplemental level of Arg is much higher than the value used by Wessels et al. (2023) (SID Arg estimated 1.10%), Luise et al. (2023), and Zhu et al. (2017), which can be estimated from reported total values and diet composition. Wessels et al. (2023) did not report the study’s power; however, they used only 72 sows, suggesting further work to replicate the results with a larger sample is warranted.

Based on this prior knowledge, the objective was to evaluate the effect of Arg supplementation above current industry levels in lactation on piglet survivability. Outcomes assessed included sow feed intake, body weight change, litter growth weight, and wean weight. The hypothesis was that increasing SID Arg from a production standard level of SID Arg 0.95% to SID Arg 1.12% would improve sow average daily feed intake (ADFI) and piglet survivability.

## Materials and methods

### General

All experimental protocols adhered to guidelines for the ethical and humane use of animals for research according to the Guide for the Care and Use of Agricultural Animals in Research and Teaching (FASS, 2010) and were approved by the Institutional Animal Care and Use Committee at Iowa State University (IACUC 24–086).

### Animals, housing, and experimental design

This study was conducted from June to August 2024 at a Midwestern commercial sow research facility. Two hundred and thirty-two PIC sows (PIC 1050, Genus, Hendersonville, TN) with an average bodyweight of 258 ± 1.3 kg upon entering the farrowing room on day 112 ± 0.2 were utilized in this study. The parity distribution ranged from parity 2–9. The sows were *Porcine Reproductive and Respiratory Syndrome* virus stable. Prior to farrowing, sows were fed a standard gestation diet according to their body condition score. When entering the farrowing room, sows were randomly assigned to one of two dietary treatments.

Sows were weighed (Mettler Toledo, Columbus, OH, USA) when entering the farrowing room and placed into farrowing stalls (1.5 × 2.1 m). Backfat (BF) was measured at the 10th rib using ultrasound (Tecnoscan SF-1, Tecnovet, Barcelona, Spain) after the sows were placed in the stalls. Body condition score (BCS) was measured at the same time on a scale of one to five, with one being underconditioned and five being over conditioned. The caliper (Pipestone, Pipestone, MN, USA) was placed at the last rib and gently placed across the back to allow for an accurate and consistent measurement of BCS. The farrowing rooms were environmentally controlled (19–24°C).

Within approximately an hour after farrowing, piglets from sows with more piglets than functional teats were moved to sows with fewer than 11–13 piglets, ensuring transfers occurred within the same dietary treatments. Piglets selected for cross-fostering were matched to the receiving litter based on body size. Whole litters were weighed post-foster 24 hours post-farrowing and again at weaning, approximately 21 days of age (Mettler Toledo, Columbus, OH, USA). Piglet mortalities and morbidities were recorded throughout the lactation period. Piglet weight and the date of removal were recorded, and the removal weight and days of nursing were incorporated into the weaning litter weight values to determine litter growth rate. Litter weights, days, and the number of piglets weaned were used to calculate piglet ADG.

### Diets and feeding

Two dietary treatments were formulated to different standardized ileal digestible (SID) Arg levels. The control diet (CON) was formulated to 0.95% SID Arg, while the experimental diet (ARG) was formulated to 1.12% SID Arg, based on the previous work by Wessels et al. (2023). Arginine was added using L-arginine (CJ Bio, Fort Dodge, IA), replacing corn, and all other ingredients were held constant. Diets were formulated to meet or exceed all other NRC (2012) recommendations. Based on the 2012 NRC model with historical farm values, the expected SID Arg: Lys ratio was 55 or the equivalent of 27.13 g of SID Arg/d when SID Arg was fed at a level of 0.43%. The ingredient composition is shown in Table 1. The calculated and analyzed nutrient composition is shown in Table 2. Sows were hand-fed the dietary treatments throughout lactation. Sows were fed a dietary treatment at 1.5 kg/d from the time they entered the farrowing room until they farrowed. On day 1 post-farrow, sows were fed 3.0 kg, 4.5 kg on day 2, 6.0 kg on day 3, and ad libitum for the remainder of lactation. Feed intake and refusal were recorded at the end of each day by collecting the remaining feed and comparing it with the known amount fed previously. Analyzed dietary Arg levels were converted to SID Arg levels and used along with ADFI to estimate sows’ SID Arg intake in g/d.

**Table 1:** Ingredient composition of experimental diets (as-fed basis) for evaluation of feeding differing levels of arginine to lactating sows.

	Dietary SID Arg, %	
Ingredient	0.95	1.12
**Corn**	73.75	73.57
**Soybean meal (46.5% crude protein)**	20.38	20.38
**Monocalcium phosphate**	1.14	1.14
**Calcium carbonate**	1.06	1.06
**Fat**	1.00	1.00
**Liquid lysine**	0.67	0.67
**Salt**	0.50	0.50
**Acidifier^b^**	0.40	0.40
**Threonine^b^**	0.33	0.33
**L-valine**	0.20	0.20
**L-arginine^c^**	0.02	0.20
**Methionine^b^**	0.16	0.16
**Sow vitamin and mineral mix^a^**	0.15	0.15
**Choline chloride 60%**	0.12	0.12
**Flow agent^b^**	0.05	0.05
**L-tryptophan**	0.05	0.05
**Vitamin D^b^**	0.03	0.03
**Total**	100.0	100.0

a Vitamin trace mineral premix; Provided 9,011 IU vitamin A, 2,254 IU Vitamin D, 60 IU vitamin E, 3.8 mg vitamin K, 7.1 mg riboflavin, 39.9 mg niacin, 2.2 mg thiamin, 1.7 mg folic acid, 0.2 mg biotin, 20 mg Cu (copper sulfate and basic copper chloride), 0.7 mg I (ethylenediamine dihydroiodide), 100 mg Fe (ferrous sulfate), 50.1 mg Mn (manganous oxide), 0.3 mg Se (sodium selenite and selenomethionine hydroxy analogue), and 120 mg Zn (zinc sulfate) per kg of the diet.

b Viglex (Provimi, Rotterdam, Netherlands), ThrPro (**CJ BIO**, Fort Dodge, IA, USA), Alimet Liquid (Novus, Chesterfield, MO, USA), Promote Defusion 501 (Provimi, Rotterdam, Netherlands), Rovimix HY-D (DSM—Firmenich, Maastricht, Netherlands).

c **CJ BIO**-America, Fort Dodge, IA.

**Table 2:** Calculated and analyzed composition of experimental diets for evaluation of feeding differing levels of arginine (Arg) to lactating sows.

	Dietary SID^a^ Arg, %	
	0.95	1.12
**Calculated Composition**		
**ME^b^, Mcal/kg^c^**	3.19	3.19
**Crude protein, %**	15.65	15.81
**Total arginine, %**	1.00	1.17
**Total lysine, %**	0.98	0.98
**Total Arg: Lys^d^**	1.02	1.20
**SID arginine**	0.95	1.12
**SID lysine**	1.05	1.05
**SID Arg: Lys^d^**	0.90	1.07
**Analyzed Composition**		
**GE^e^, Mcal/kg**	3.85	3.82
**Crude protein, %**	14.40	15.11
**Total arginine, %**	0.95	1.17
**Total lysine, %**	1.05	1.15
**Total Arg: Lys^d^**	0.90	1.02

a Standardized ileal digestibility.

b Metabolizable Energy.

c Megacalorie per kilogram.

d Lysine.

e Gross Energy.

#### Analysis

##### Diet analysis

Feed samples were taken from the feed mill of each batch of both diets. Samples were homogenized and ground through a 1 mm screen. Diet samples were ground through a 1 mm screen using a Wiley Mill (Variable Speed Digital ED-5 Wiley Mill; Thomas Scientific, Swedesboro, NJ). Ground samples were submitted to the University of Missouri Agricultural Experimental Station Laboratories (Columbia, MO) for complete amino acid profiling using cation-exchange chromatography coupled with post-column ninhydrin derivatization and quantification (method 982.30 E and 988.15; AOAC, 2006). Feed samples were also analyzed for gross energy at Iowa State University using an isoperibolic bomb calorimeter (model 6200; Parr Instruments Co., Moline, IL). The analyzed nutrient composition of the experimental diets is shown in Table 2.

##### Statistical analysis

Data were analyzed using generalized mixed linear models, with treatment as a fixed effect and group as a random effect. The group was the farrowing batch. Post-foster litter size and post-foster litter weights were added to models for the number of piglets weaned, litter wean weight, livability, mortality, litter ADG, and piglet ADG. Models were fit and data analyzed using R (v4.4.1; Posit team, 2024). Results were considered significant at *P* ≤ 0.05 and a tendency at 0.50 ≤ *P* ≤ 0.10. Studentized residuals were used to detect outliers greater than 3 standard deviations from the mean; a total of 16 observations were removed, with 10 from the ARG and 6 from the CON. Assumptions of all models were verified using residual plots. The sow and her litter were considered the experimental unit.

## Results

### Sow performance

Sow performance data are presented in Table 3. Sows entered the farrowing room at an average back fat of 10.07 ± 0.282 mm. There was no difference in sow BF between treatments at weaning. Body condition score at entry averaged 3.0 ± 0.06. There was no difference between BCS at wean. Body weight change from farrow to wean was not different. Sow lactation length averaged 20.0 ± 0.11 days. Sow ADFI also did not differ between treatments. Sow SID Arg intake did differ between the two treatments, SID Arg 61.2 ± 0.987 g/d and 83.7 ± 0.987 g/d (*P* < 0.01).

**Table 3:** Effects on sow performance for evaluation of feeding differing levels of arginine (Arg) to lactating sows.

	Dietary Treatment SID Arg, %			
Parameter	0.95	1.12	SEM^a^	P-value
**Parity**	4.50	4.30	0.241	0.47
**Lactation length, d**	20.0	20.0	0.113	0.76
**SID^b^ Arg intake, g/d^c^**	61.2	83.7	0.99	<0.01
**Average daily feed intake^d^, kg/d**	7.28	7.48	0.09	0.12
**Feed intake, kg**	145.83	149.44	2.22	0.25
**Bodyweight entry, kg**	257.88	259.00	1.843	0.67
**Bodyweight post-farrow, kg**	232.26	232.90	2.991	0.79
**Bodyweight wean, kg**	242.39	240.64	2.522	0.63
**Bodyweight change, kg**	10.10	7.90	1.590	0.32
**Bodyweight change, %**	4.24	3.38	0.692	0.38
**Backfat entry, mm**	10.13	10.00	0.282	0.71
**Backfat wean, mm**	9.57	9.39	0.254	0.79
**Backfat change, mm**	−0.56	−0.66	0.188	0.53
**Body condition score entry**	2.94	3.01	0.058	0.28
**Body condition score wean**	2.72	2.76	0.066	0 .57

a Standard error of the mean.

b Standardized ileal digestibility.

c Calculated using sow feed intake and analyzed dietary Arg levels.

d Average daily feed intake from entering the farrowing room to wean.

### Piglet performance

The number of piglets per sow post-foster was CON 13.20 ± 0.209 and ARG 14.20 ± 0.209 (*P* < 0.01) (Table 4). The number of piglets weaned did not differ significantly between sows from both dietary treatments, weaning 12.10 ± 0.119 piglets. Starting litter weights were 20.07 kg (CON) and 21.66 kg (ARG) (*P* < 0.01). At weaning, litter weights were not different. Piglet average daily gain (ADG) did significantly differ between the two treatments. Piglet ADG did significantly differ between the two treatments. The CON had a piglet ADG of 0.25 kg/d, and the ARG had a piglet ADG of 0.24 kg/d (*P* = 0.03). Litter ADG, however, did not differ between the dietary treatments. Total livability as a percent did not differ significantly (*P* = 0.29), with livability for CON at 93.3% and for ARG at 94.0%. Post-foster mortality also did not differ, was 6.72% and 596%, respectively.

**Table 4:** Effects on piglet performance for evaluation of feeding differing levels of arginine (Arg) to lactating sows.

	Dietary Treatment			
	SID^a^ Arg, %			
Parameter	0.95	1.12	SEM^b^	P-value
**Total born, n**	17.00	17.30	0.421	0.63
**Born alive, n**	14.60	14.70	0.373	0.88
**Post-foster litter size, n**	13.20	14.20	0.209	<0.01
**Number weaned, n^c^**	12.10	12.10	0.119	0.79
**Starting litter weight, kg**	20.07	21.66	0.357	<0.01
**Litter wean weight, kg^d^**	79.97	79.46	1.190	0.69
**Piglet average daily gain, kg^c^**	0.25	0.24	0.004	0.03
**Litter average daily gain, kg^d^**	2.93	2.97	0.045	0.54
**Livability, %**	93.60	94.00	0.008	0.43
**Mortality, %**	6.72	5.96	0.008	0.43

a Standardized ileal digestibility.

b Standard error of the mean.

c Number of piglets post-foster included as a covariate.

d Starting litter weight included as a covariate.

## Discussion

In the present study, increasing dietary SID Arg to a calculated 1.12% did not affect sow body weight, feed intake, litter wean weight, or piglet survivability. However, the number of sows available to be included in this study was limited due to financial constraints, and a post-hoc analysis (*α* = .05, 80% power) indicated that the study had very low power, approximately 4%, to detect the observed difference in livability between treatments (93.3 vs 94.0%). These results suggest that the study was underpowered to detect small, yet potentially biologically relevant, differences in livability and mortality. In addition, due to the ownership of the sow farm at which this study was conducted changing, the study was unable to follow the sows to the next farrow to receive wean-to-estrus data and sow performance or litter performance at the next farrow.

Arginine serves as a precursor for nitric oxide and polyamine synthesis, contributing to angiogenesis and cell proliferation (Wu et al. 2013). Due to these roles and the inefficiency of a young pig’s ability to synthesize Arg endogenously, Arg is essential in young pigs (Wu 1997; Kim and Wu 2004; Wu et al. 2004). However, meeting these needs can be challenging. The modern hyperprolific sow is often in a catabolic state during lactation (Wessels et al. 2023). A catabolic state means that the nutritional demands of lactation exceed what the sow can consume, causing her to draw on her own body stores and lose weight, resulting in decreased performance, such as lower wean weights and longer wean-to-estrus intervals, and potentially limiting Arg availability to piglets.

Previous research evaluating Arg supplementation during lactation has reported inconsistent effects on sow and piglet performance. Wessels et al. (2023) observed improvements in piglet growth, whereas Holanda et al. (2019) reported no response at higher SID Arg levels. This inconsistency may be due to fluctuation in the dietary SID levels of Arg used in previous work. With Wessels et al. (2023) supplementing at an estimated SID Arg level of 1.10% and Holanda et al. (2019) using a basal diet that had a higher SID Arg level of 1.22% and supplementing with SID Arg 2.15%.

Mateo et al. (2008) supplemented sows with 1% L-arginine HCl (SID Arg estimated at 2.12%) throughout gestation and lactation and observed increased concentrations of total amino acids in sows’ milk, including Arg, but more notably glutamate and proline. This pattern suggests that additional dietary Arg may be catabolized by mammary gland arginase, generating glutamate and proline. In the present study, milk protein was not analyzed; however, if Arg supplementation improved milk composition, then piglet weights would be expected to improve, as reported by Mateo et al. (2008). This indicates that the amount of AID Arg (1.12%) supplemented was potentially insufficient to affect the sow’s milk composition or improve piglet performance.

Arginine’s role in vasodilation and body growth through nitric oxide synthesis and polyamine production could contribute to improved livability in pre-wean pigs. Improved vascular function may improve thermoregulation and nutrient deposition in tissue, potentially contributing to improved piglet survival. Piglets experience rapid growth, yet their endogenous Arg synthesis is limited (Wu et al. 2007). Previous work has demonstrated that 14- to 21-day-old piglets have a reduced capacity to synthesize citrulline compared to older pigs, which, in turn, limits endogenous Arg synthesis (Wu et al. 2007). While supplementing Arg to the sow could theoretically offset this limitation by increased Arg availability to the litters, this does not appear to be the mechanism. Instead, Mateo et al. (2008) demonstrated increases in glutamate and proline in milk protein from Arg-supplemented sows. Suggesting that improvements to piglet performance may not be driven by increased Arg in milk protein, but by improved concentrations of Arg precursors, such as glutamine and proline. However, in the present study, piglet mortality and livability were not affected, consistent with previous reports (Holanda et al. 2019; Wessels et al. 2023), indicating that Arg supplementation during lactation does not consistently improve survivability.

Consistent with previous work, Arg supplementation did not impact sow feed intake (Mateo et al. 2008; Zhu et al. 2017). Gao et al. (2020) demonstrated that feed intake reached a dose-dependent plateau when Arg was fed up to a total Arg: lysine ratio of 1.01. In the present study, there was no difference between the two treatments ADFI with a total Arg: Lys ratio of 0.96, which is below the plateau reported by Gao et al. (2020). The lack of response in other performance metrics in the present study could also be explained by the other amino acids in the diet. The Arg to lysine ratio was set at total 1.02 in the CON and 1.20 in the ARG (Table 2), but analysis revealed it was lower: 0.81 in the CON and 0.96 in the ARG.

In the current study, SID Arg 1.12% had no effect on sow weight, feed intake, litter wean weight, or piglet survivability. These results did not provide sufficient evidence to support the hypothesis that sow and piglet performance would improve when calculated SID Arg is increased from 0.95% to 1.12%.

## List of abbreviations

AA: Amino Acid
ADG: Average Daily Gain
ADFI: Average Daily Feed Intake
BCS: Body Condition Score
BF: Backfat
BW: Bodyweight
IUGR: Intrauterine growth restriction
SID: Standard Ileal Digestible
